# A change in the NICE guidelines on antibiotic prophylaxis: British Heart Valve Society update

**DOI:** 10.3399/bjgpopen17X100593

**Published:** 2017-03-15

**Authors:** John B Chambers, Martin H Thornhill, Mark Dyer, David Shanson

**Affiliations:** 1 Consultant Cardiologist and Professor of Clinical Cardiology, Guy's and St Thomas' Hospitals, London, UK; 2 Professor of Translational Research in Dentistry, The School of Clinical Dentistry, University of Sheffield, Sheffield, UK; 3 Consultant Cardiologist, Taunton and Somerset NHS Trust, Taunton, UK; 4 Honorary Consultant Microbiologist, Great Ormond Street Hospital for Children, London, UK

**Keywords:** endocarditis, antibiotic prophylaxis, dental procedures

## Introduction

The National Institute for Health and Care Excellence (NICE) has made an important change to Clinical Guideline 64 (CG64)^1^ adding the word ‘routinely’ to Recommendation 1.1.3: 'Antibiotic prophylaxis against infective endocarditis is not recommended routinely for people undergoing dental procedures.' In a letter about the change to the MP Chris Philp,^2^ Sir Andrew Dillon, CEO of NICE, confirmed that '… in individual cases, antibiotic prophylaxis may be appropriate.'

## Why has this change occurred?

This change followed approaches to Sir Andrew Dillon by Chris Philp and the widow of a patient with a replacement aortic valve who died from infe ctive endocarditis (IE) developing after unprotected dental scaling. Their case included:

1. **Evidence that antibiotic prophylaxis is effective in people at high risk of IE having high risk dental procedures** (Box 1). There are no randomised controlled trials. However, a French community study^4^ showed a 14-fold higher incidence of IE in people with replacement valves having unprotected dental procedures (1/10 700) compared with protected procedures (1/149 000). Horstkotte^5^ found no cases of IE in 229 people with replacement valves having protected dental procedures compared with 2 of 117 (1.7%) having unprotected procedures. A recent retrospective analysis of hospital-acquired data in the UK^6^ suggested that 277 patients at all levels of risk needed to have antibiotic prophylaxis to prevent one episode of IE.
2. **The observed increase in the incidence of IE in the UK since the original 2008 NICE guidance was introduced**. The risk of IE has been increasing constantly in the US and Europe. However, an interrupted time sequence study^6^ showed that this rate accelerated in the UK after 2008 with an estimated 35 extra cases of IE every month 5 years after the change above the expected number based on the pre-2008 incidence. This was associated with an 88% fall in prescriptions of antibiotics.
3. **Limitations of the NICE process.^3^** The NICE process is well-designed for its original purpose of making decisions about the cost-effectiveness of drug therapies or procedures for which randomised controlled trial data are available. However, it is not appropriate for questions of clinical judgement for which other types of evidence exist. NICE assessed only a small proportion of the available evidence and made a number of errors of interpretation including underestimating the risk of patients with prosthetic valves developing IE and underestimating the bacteraemia arising as a result of invasive dental procedures.^3^ NICE grossly overestimated the risks of antibiotic proyhphylaxis and this contributed to a decision that antibiotic prophylaxis was not cost-effective. In fact adverse effects from oral amoxicillin prophylaxis are uncommon with no deaths reported in Europe since records began^7^ although clindamicin has a slightly higher level of risk.^8^ Both are cost-effective in high-risk patients having high-risk dental procedures.^8^
4. **A change in the law of consent.** ^9–11^ It is now necessary for dentists to appraise their patient of the differences between NICE and other guidelines if it is likely that they would have a special interest, for example, if they have a replacement heart valve or prior IE.^12^ The patient should then be allowed to make up their own mind whether or not to have antibiotic prophylaxis. General Medical Council/General Dental Council standards and the advice of the medical/dental defence organisations highlight the need for this discussion (and the patient’s decision) to be recorded in the clinical records.

**Box 1.** Summary of guidance

People at high risk: replacement heart valves or prior endocarditis People at moderate risk: native valve disease High-risk dental procedure: extractions, root canal treatment, dental scaling and other procedures involving manipulation of the gums Antibiotic prophylaxis: indicated for people at high-risk having high-risk dental procedures. Record details of consent process in the dental notes. Use amoxicillin 3 g or clindamicin 600 mg orally 1 hour before Other advice: dental surveillance every 6 months (high-risk people) or annually (medium-risk people), avoid tattoos and intravenous drug use Warning: consider infective endocarditis with persistent fever or night sweats especially with systemic symptoms. Take at least two sets of blood cultures before starting an antibiotic course.

## Is IE caused by poor oral hygiene or dental extractions?

Streptococci account for approximately 40% of cases of IE. There is a tendency to regard the mechanism by which these cause IE as either poor dental hygiene or dental procedures as if these are mutually exclusive. This misunderstanding probably underlies some of the opposition to targeted antibiotic prophylaxis shown by NICE and other commentators. In fact, both mechanisms are likely to contribute. In a recent study,^13^ 68 patients had IE thought to be of oral origin, 60 attributed to poor oral health and eight to dental procedures. The larger proportion of these cases could be prevented by improving dental care, but the smaller proportion would still require antibiotic prophylaxis before the procedure. The person whose death from IE led to the recent approach to NICE had good oral hygiene.

## How does this affect clinical practice?

Although the dentist is responsible for consent, the patient will have to be made aware if they are at high-risk of IE and this is the prime responsibility of a cardiologist specialising in valve disease. Patients under continuing cardiac surveillance should expect to receive education,^14^ and a summary of advice about antibiotic prophylaxis in a letter which can be shown to their dentist. However, many patients with prior endocarditis or replacement valves are cared for solely in the community,^15^ and for these, the dentist should consider seeking advice from a cardiologist. Sometimes the dentist refuses a patient’s request to have antibiotic prophylaxis although this may become less frequent with the latest modification of NICE guidance. GPs may then be asked to intercede and are often willing to issue a prescription, but otherwise, should seek advice from a cardiologist. Prophylaxis for adults should be with amoxicillin 3 g orally 1 hour before the procedure or, for patients with penicillin hypersensitivity, clindamicin 600 mg orally (Box 1).

Staff in general practices should emphasise that good oral hygiene and regular dental review are as important as antibiotic prophylaxis, if not more so, in reducing the risk of IE.^7^ The European Society of Cardiology recommend^12^ strict dental and cutaneous hygiene with regular dental surveillance (Box 1). It is also important to educate patients at moderate and high risk in recognising the possibility of IE. Typically, there may be persistent night sweats, general malaise, and weight loss. At least two sets of blood cultures should be taken before starting antibiotics. The British Heart Foundation produce warning cards that can be given to patients.^16^

The subtle change makes NICE guidance less dogmatic and allows clinicians to use their clinical judgment, follow well-accepted international guidelines,^12^ and provide the care their patients want.
